# A psychological symptom based machine learning model for clinical evaluation of irritable bowel syndrome

**DOI:** 10.12688/openreseurope.15009.1

**Published:** 2023-01-27

**Authors:** Noman Haleem, Astri J. Lundervold, Gülen Arslan Lied, Eline Margrete Randulff Hillestad, Maja Bjorkevoll, Ben René Bjørsvik, Erica Sande Teige, Ingeborg Brønstad, Elisabeth Kjelsvik Steinsvik, Bharath Halandur Nagaraja, Trygve Hausken, Birgitte Berentsen, Arvid Lundervold

**Affiliations:** 1 National Center for Functional Gastrointestinal Disorders, Department of Medicine, Haukeland University Hospital, Bergen, Norway; 2 Mohn Medical Imaging and Visualization Center, Department of Radiology, Haukeland University Hospital, Bergen, Norway; 3 Department of Biological and Medical Psychology, University of Bergen, Bergen, Norway; 4 Center for Nutrition, Department of Clinical Medicine, University of Bergen, Bergen, Norway; 5 Center of International Health, Faculty of Medicine, University of Bergen, Bergen, Norway; 6 Department of Clinical Medicine, University of Bergen, Bergen, Norway; 7 National Center for Ultrasound in Gastroenterology, Department of Medicine, Haukeland University Hospital, Bergen, Norway; 8 Department of Biomedicine, University of Bergen, Bergen, Norway

**Keywords:** Irritable bowel syndrome, anxiety, fatigue, machine learning, classification

## Abstract

**Background**: Irritable bowel syndrome (IBS) is a chronic functional gastrointestinal disorder characterized by recurrent abdominal pain associated with alterations  in stool form and/or stool frequency. Co-morbidities such as anxiety, depression, fatigue, and insomnia are frequently reported by patients suffering from IBS. Identification of these symptoms should thus be an integral part of an IBS assessment.      However, an optimal tool to screen for core psychological symptoms in IBS is still  missing. Here, we aim to develop a psychological symptom based machine learning model to efficiently help clinicians to identify patients suffering from IBS.

**Methods**: We developed a machine learning workflow to select the most significant psychological features associated with IBS in a dataset including 49 patients with IBS and 35 healthy controls. These features were used to train three different types of machine learning models: logistic regression, decision trees and support vector machine classifiers; which were validated on a holdout validation dataset and an unseen test set. The performance of these models was compared in terms of balanced accuracy scores.

**Results**: A logistic regression model including a combination of symptom features associated with anxiety and fatigue resulted in a balanced accuracy score of 0.93 (0.81-1.0) on unseen test data and outperformed the other comparable models. The same model correctly identified all patients with IBS in a test set (recall score 1) and misclassified one non-IBS subject (precision score 0.91). A complementary post-hoc leave-one-out cross validation analysis including the same symptom features showed similar, but slightly inferior results (balanced accuracy 0.84, recall 0.88, precision 0.86).

**Conclusions**: Inclusion of machine learning based psychological evaluation can complement and improve existing clinical procedure for diagnosis of IBS.

## Introduction

Irritable bowel syndrome (IBS) is a disorder of gut-brain interaction characterized by recurring abdominal pain associated with alterations in stool form and/or stool frequency ^1, 2^. Moreover, psychological co-morbidities are frequently reported by patients with IBS ^3^, including symptoms associated with depression ^4^, anxiety ^5^ and disturbed sleeping patterns ^6^. These symptoms have been related to the gastrointestinal (GI) symptoms of IBS ^7– 9^ as well as reduced activity and productivity at work ^10– 12^.

With characteristics extending beyond GI symptoms, IBS is viewed as a multi-faceted disorder, where inclusion of psychological factors in a clinical assessment procedure is shown to improve identification of patients with IBS ^13, 14^. A systems view of IBS ^15^ is in line with the so-called brain-gut axis, describing a set of bi-directional communication pathways between the brain and the gut that are modulated by the gut microbiome through neuronal signaling and systemic circulatory mechanisms ^15, 16^. This brain-gut axis is viewed as the underlying link between psychological and GI health as its dysfunction caused by e.g. altered microbial composition is suspected to affect brain structure ^17^ and function ^18^, eventually leading to psychological disturbances.

The concept of brain-gut axis is still in its infancy from an experimental perspective ^19^. The high prevalence of psychological symptoms in patients with IBS immediately calls for tools that can contribute to an improved assessment procedure. A definite diagnosis of IBS is challenging even for expert clinicians due to a significant symptom overlap with normal function and with other functional or organic GI disorders. Today, an IBS diagnosis is primarily based on assessment of GI symptoms and exclusion of organic causes ^20, 21^. With increasing awareness of the importance of psychological symptoms, there are strong arguments for including evaluation of psychological symptoms as an integral part of the assessment procedure when diagnosing patients with IBS ^4, 22, 23^. With a more holistic approach including both psychological and somatic symptoms, an improved clinical management and treatment of patients with IBS should be expected ^10, 24^.

Despite improved awareness of the importance of psychological symptoms, there is still no clear consensus on which symptoms to assess in order to more precisely identify a patient with IBS. A suitable psychological evaluation system that can help the clinicians in making diagnostic decisions does not yet exist. This unmet need could be attributed to limited knowledge about (i) selection of suitable questionnaires for psychological evaluation of IBS, (ii) how much weight should be assigned to different items in these questionnaires, (iii) what should be optimal cut off values and (iv) how these values can be aggregated into final scores to identify core psychological symptoms in IBS.

The presented study aims to develop a psychological evaluation system for clinical diagnosis of IBS by using a data driven approach based on machine learning methods. A feature selection workflow based on three different types of machine learning models was used to identify a combination of the most significant psychological symptoms from a dataset of four relevant questionnaires. We used this symptom combination to develop a model for classifying patients with IBS and healthy controls (HC) in an unseen test set. Our new evaluation model is likely to improve awareness of IBS-related psychological symptoms and thus contribute towards improvement of traditional IBS diagnosis and management in clinical practice.

## Methods

### Data collection and management

The dataset used in this study comprised of the following three questionnaires: the Hospital Anxiety and Depression Scale (HADS) ^25^, the Chalder Fatigue Scale (CFS) ^26^ and the Bergen Insomnia Scale (BIS) ^27^. These questionnaires were filled in by the participants of the Bergen Brain Gut study (Trial registration number NCT04296552, registered on 4th March, 2020) and the details of inclusion and exclusion criteria are provided in the study protocol paper ^28^.

HADS is a validated questionnaire to screen for anxiety and depression in primary, somatic, and psychiatric care. The questionnaire comprises of 14 items, which are filled by participants with respect to their behaviours and feelings during the last week. Each question carries a maximum score of three, resulting in a total maximum possible score of 42. The odd numbered items in the questionnaire measure anxiety and even numbered items measure depression status in the participants.

The CFQ is a validated self-rated fatigue scale designed to detect cases, assess fatigue severity and change in level of fatigue over time in adults. The CFQ scale gives measures of mental and physical fatigue. It comprises of a total of 11 items and uses two types of scoring systems i.e., bimodal and Likert scoring. In the bimodal scoring system, respondents answer each question with a 0 or a 1 to indicate whether the questions apply to them or not. In the Likert scoring system, respondents assign a score of 0 to 3 to indicate how each statement applies to them, from “less than usual” to “much more than usual”.

BIS questionnaire is based on the diagnostic criteria for insomnia as described by the Diagnostic and Statistical Manual of Mental Disorders (DSM-IV). The six items on BIS are rated on an 8 point scale, ranging from 0 to 7 days per week during the last month. Sleep impairment is assessed by the first four items (criteria A of the DSM-IV) and the last two items refer to daytime sleepiness / tiredness and dissatisfaction with sleep (criteria B). A DSM-IV diagnosis of insomnia is fulfilled if a respondent reports equal to or more than 3 days per week on at least one of the A items and equal to or more than 3 days per week on at least one of the B items. In addition, a total composite score is calculated by adding together the scores for each item, with a possible range from 0 to 42.

A total of 84 individuals (including male and female participants) were included in the presented study: 49 patients with IBS (constipation, diarrhea and mixed) and 35 healthy controls. All participants filled out HADS questionnaire within an online patient survey system (Check-Ware, Check-Ware AS, Trondheim, Norway), whereas the CFS and BIS questionnaires were filled out on paper templates and later digitally entered into a FileMaker Pro relational database (version 19, Claris International Inc.).

All three questionnaires were retrieved from their source databases and concatenated to a single data frame, a two-dimensional table of size n p using the Pandas data analysis and manipulation tool (version 1.3.5), using Python programming language (version 3.7). The number of participants was n = 84 and number of variables was p = 58. Each row in the data frame was indexed by participant ID after anonymisation and the other columns consisted of questionnaire data (both single item responses and total scores), age (years), gender (male=0, female=1), and disease status (HC=0, IBS=1) of the participant. The total number of variables for the HADS, CFS and BIS questionnaires was 31 (14 single items), 15 (13 items), and 8 (6 items), respectively.

The HADS questionnaire was split into an anxiety and a depression subscale to achieve a fine grained representation of both psychological symptom clusters. The anxiety subscale (ANX) was obtained by responses on the seven odd numbered questions of HADS. Similarly, the even numbered questions were included in the depression subscale (DEP).

The participants’ responses to various questions in the CFS questionnaire were text based, e.g., “Do you have problems with tiredness?” could be responded with (i) much more than usual, (ii) more than usual, (iii) no more than usual, (iv) less than usual, and (v) not at all. These textual responses were transformed to suitable numerical representations at ordinal scale i.e. integers from 1 to 5, where the upper end of the range (5) denoted symptom extremity i.e. ‘much more than usual’ and lower end (1) denoted symptom minimum i.e. ‘not at all’.

### Data preprocessing handling of missing values

The data set contained various missing values due to (i) no response of a participant for a certain questionnaire, i.e., drop out during the course of the study (ii) missing response of the participants for some questions either intentionally or by mistake and (iii) human error in data collection and entry phases. A two level approach was proposed to handle missing values. Firstly, all those participants, whose missing data proportion was above a predefined threshold T , were excluded from the analysis. The threshold T was calculated as proportion of half of the questions in the shortest questionnaire in the dataset to the total number of questions in all four questionnaires, which resulted in T = 9%. Secondly, the ‘most frequent value’ data imputation method was applied for all those participants who were missing less than 9% of their total data. The imputation method assigned the most frequently occurring value for each variable to any missing values within that column of the data frame. Prior to this assignment, the dataset was split in IBS and HC subsets, so that any missing value for a participant with IBS could only be imputed by the most frequently occurring value within the IBS group and vice versa.

### Stratified sampling of participants into train, validation and test sets

In order to develop and subsequently test machine learning models, the imputed dataset was divided into training, validation and test sets in proportion of 60%, 20% and 20%, respectively. The training set was used for developing the models, the validation set was held out for selection of the best model and the test set served as a proxy of unseen data to test model performance in real world scenarios. As the patient cohort consisted of various IBS subcategories and both genders, a stratified approach was used for sampling to ensure optimal representation of underlying participant population within each data subset. For this purpose, all participants in the study were first arranged into seven subgroups based on all possible combinations of their gender and IBS subtype, except male participants with constipation type IBS as no subject in patient cohort belonged to this category. This was followed by randomized sampling (with fixed seed) of the participants from each subgroup using the StratifiedShuffleSplit function in the scikit-learn machine learning library (version 1.0.2) into training, validation and test sets ^25^. It is important to note that stratified sampling approach evenly distributed participant population (class labels) in all three sets without affecting overall sample size.

### Feature selection

**Figure 1.**
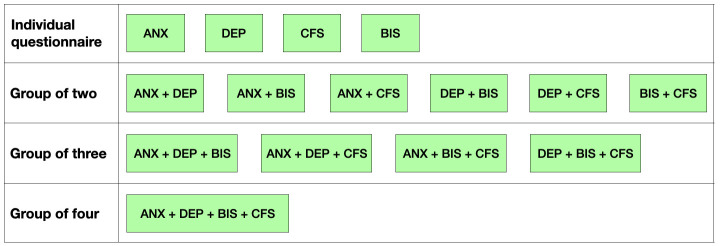
Various pools of features generated by arranging four questionnaires into all possible combinations. ANX = anxiety questionnaire; DEP = depression questionnaire; BIS = Bergen Insomnia Scale; CFS = Chalder Fatigue Scale.

A wrapper type feature selection method i.e. sequential backward feature selection (SBFS) technique was used to select most significant features by excluding redundant and non-contributing features and to reduce dimensionality in the dataset. The SBFS method worked through iterative removal of features from a given input feature set based on cross-validation score of an estimator to achieve a final feature vector of a certain predefined length. This process essentially required defining at least three factors namely (i) input feature set, (ii) length of final feature vector and (iii) type of estimator (or model). The values of these factors were unknown a priori as the most significant feature vector may potentially come from any combination of questionnaires, any number of features and any type of estimator. Hence, a three-pronged approach was proposed for feature selection. Firstly, all four questionnaires were arranged in all possible combinations at four different levels i.e. (i) single, (ii) group of two, (iii) group of three and (iv) group of four questionnaires, resulting in fifteen groups of questionnaires, as shown in Figure 1. The participant age and encoded gender were also added to these groups of questionnaires, which were then used as input feature sets for SBFS technique.

**Figure 2.**
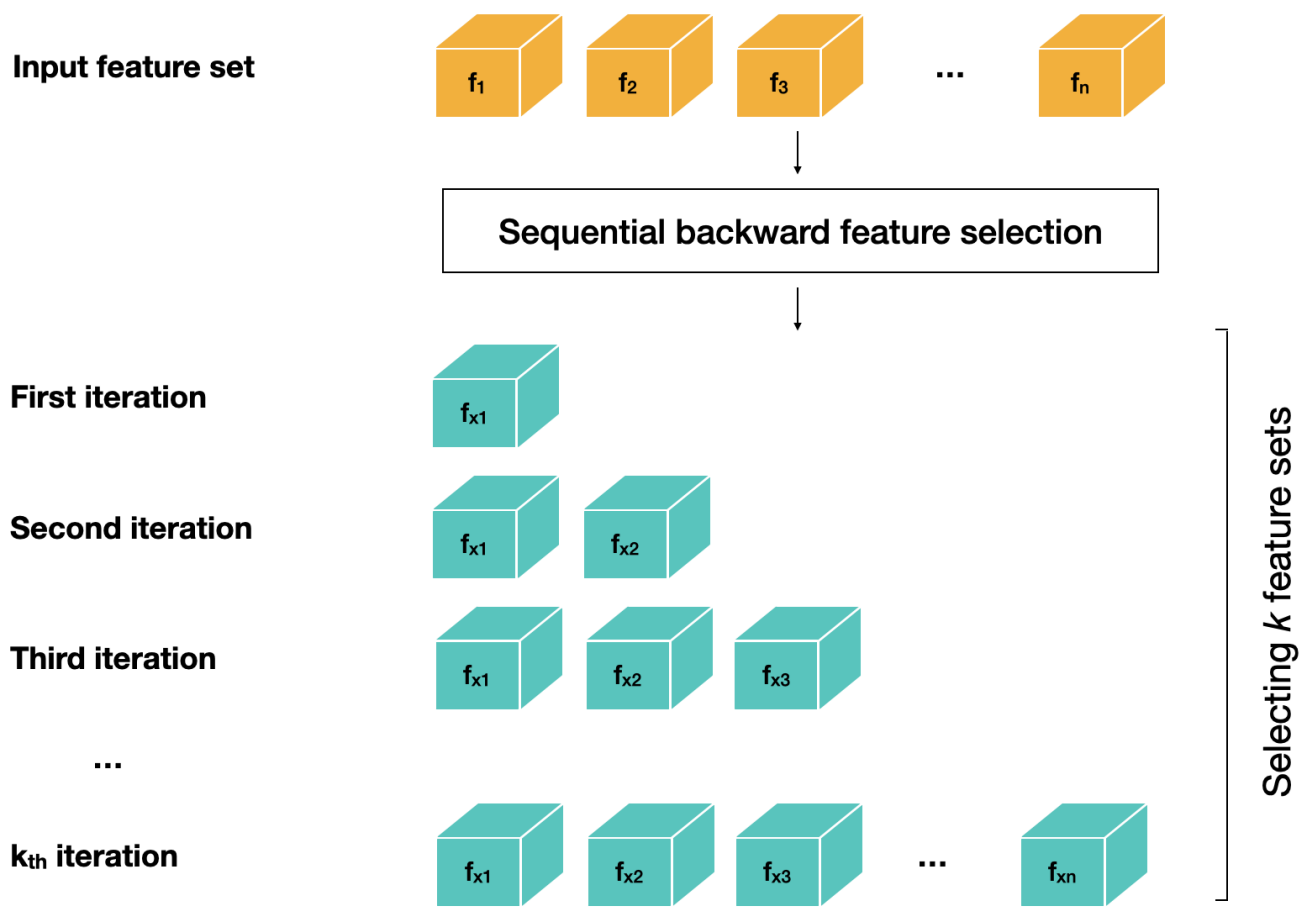
Sequential backward feature selection from input feature set.

Secondly, the SBFS algorithm was programmed to iteratively select k feature vectors from each questionnaire group, where k = 1, . . . , N and N is the total number of features within that feature set, as schematically shown in Figure 2. Thirdly, three different types of machine learning models were used as estimators, i.e., logistic regression (LR), support vector machine classifier (SVC) and decision trees classifier (DT). The hyper parameters for all estimators were kept at default values as provided in scikit-learn library functions. A ten-fold stratified cross validation type evaluation metric was used in feature selection, where each fold contained a stratified sample from the training set, as described in Stratified sampling of participants into train, validation and test sets. Balanced accuracy score was used as an evaluation metric in feature selection process.

### Development and validation of machine learning models

The feature selection process generated 882 feature vectors for 15 questionnaire groups and three model types, i.e., each model selected 294 feature vectors. Some of these vectors were not valid representation of all participating questionnaires in a group as they did not include features from all of the questionnaires. Such feature vectors were excluded from further analysis on grounds of non-representativeness. The remaining feature vectors were used to train the same type of machine learning model that was used in their selection process e.g. the feature vectors selected using logistic regression model were used to develop logistic regression type models using the training set.

The best performing models in each group of questionnaires for each model type were selected by applying those models on the holdout validation data set. The models resulting in highest balanced accuracy score on validation data were nominated as the best performing models within that group of questionnaires. If any two or more models scored equally in validation phase, the model with highest cross validation accuracy score in training phase was preferred over the others. This model selection phase resulted in 15 models for each model type i.e. one model for each group of questionnaires.

The best performing model for each model type was then selected on the basis of the highest score on validation dataset. This resulted in three final models. These models were once again trained, this time using both training and validation data sets and applied on the unseen test set for the final evaluation of their predictive performances. The outcomes of the models were evaluated through accuracy score, balanced accuracy score, recall, precision, F-score and AUROC score i.e. area under the receiver operating characteristic (ROC) curve. The best model among the final three models was selected on basis of highest balanced accuracy score and the same model was nominated as the final model that can be applied for psychological symptom-based evaluation of patients with IBS.

The contribution of the constituents of the input feature set in the best performing model was evaluated and ranked using the permutation feature importance method ^29^. Permutation feature importance works by measuring the decrease in model score by randomly shuffling the values of a single feature at a time, thus breaking the relation between the feature and the corresponding target (i.e., HC or IBS), and thereby assessing how important a given feature is for the performance of this particular model. The significance of the feature set associated with the best model and level of classification performance was also tested using post-hoc Leave-One-Out Cross validation (LOOCV) analysis, which was applied once and in turn to each of 84 instances in the dataset. In each LOOCV iteration, the selected instance was kept out as a single item test set, whereas all remaining 83 instances were used for training the model.

## Results

### Participant characteristics

The key characteristics of the participant cohort including demographics (age and gender) and total scores from patient questionnaires are given in Table 1. The p-values (where applicable) are determined using independent Student’s T -test.

**Table 1.** Key characteristics of the participants included in the study. IBS = Irritable bowel syndrome; HC = Healthy control group; TFS = Total fatigue score as determined by Chalder fatigue scale; TSS = Total sleep score as determined by Bergen insomnia scale; TAS = Total anxiety score as determined by Anxiety subscale; TDS = Total depression score as determined by Depression subscale.

Variable	IBS	HC	p-values
Participant count (%)	49 (58.33)	35 (41.67)	
Males (%)	11 (50.0)	11 (50.0)	
Females (%)	38 (61.29)	24 (38.71)	
Mean age [year] (SD)	36.45 (10.91)	35.89 (12.66)	0.828
TFS (Chandler 0/1) (SD)	6.37 (3.39)	1.60 (2.46)	< 0.001
TSS (BIS) (SD)	16.92 ( 6.78)	10.29 (7.15)	< 0.001
TAS (HADS odd) (SD)	8.20 (4.11)	4.09 (3.25)	< 0.001
TDS (HADS even) (SD)	4.90 (3.09)	2.09 (2.30)	< 0.001

An imbalance in the dataset for both the IBS and HC categories and gender can be noticed as the number of both IBS and female participants are higher than HC and male participants, respectively. The mean ages for both IBS and HC groups are 35.89 and 36.45 years, respectively (p = 0.843). The total scores for fatigue, sleep, anxiety and depression are significantly higher for the IBS group compared to the HC group, as shown by their low p-values (< 0.001), although an overlap among the underlying distributions can be noticed in terms of standard deviation of these scores. The patient questionnaire dataset was split into train, validation (or valid) and test sets using shuffled stratified splitting approach as shown in Table 2.

**Table 2.** Stratified splitting of participant dataset into train, validation and test sets based on gender and IBS subtypes (IBS-C constipation, IBS-D diarrhea and IBS-M mixed). F = Female; M = Male; HC = Healthy Control.

Stratification	Total	Train set	Validation set	Test set
Constipation_F	5	3	1	1
Diarrhea_F	13	7	3	3
Diarrhea_M	6	4	1	1
Mixed_F	20	12	4	4
Mixed_M	5	3	1	1
HC_F	24	14	5	5
HC_M	11	7	2	2

### Performance of machine learning models

The feature selection workflow resulted in 263 feature vectors using LR model, 241 using DT model and 226 using SVC model, as described in Feature selection. Each of these feature vectors was used to train the same type of the model used in their selection and applied on validation dataset to analyse their performance. The balanced accuracy scores of best performing models in each questionnaire category are given in Table 3. The best performing models for each model type are shown with bold highlights in Table 3.

**Table 3.** A comparison of balanced accuracy scores of best in category models on validation dataset. ANX = Anxiety subscale; DEP = Depression subscale; BIS = Bergen Insomnia Scale; CFS = Chalder Fatigue Scale.

Questionnaire category	Logistic regression	Decision tree	Support vector machine
ANX	0.71	0.85	0.83
DEP	0.74	0.81	0.86
BIS	0.71	0.69	0.81
CSF	0.76	0.76	0.81
ANX_DEP	0.83	0.85	0.86
ANX_BIS	0.81	**0.88**	0.81
ANX_CFS	**0.86**	0.76	0.81
DEP_BIS	0.71	0.76	0.79
DEP_CFS	0.81	0.76	0.86
BIS_CFS	0.76	0.76	0.81
ANX_DEP_BIS	0.81	0.83	0.86
ANX_DEP_CFS	0.81	0.71	0.88
ANX_BIS_CFS	0.81	0.81	0.81
DEP_BIS_CFS	0.76	0.79	0.86
ANX_DEP_BIS_CFS	0.83	0.81	**0.93**

### A model for evaluation of psychological symptoms associated with IBS

The three best performing models ( Table 3) were applied on unseen test set, which was used as a proxy of real world situations to assess generalisation of the model. Various evaluation metrics from this test are reported in Table 4, where the best performing metrics are shown with bold highlights.

**Table 4.** Performance metrics for best in category models by applying on test dataset (n = 17). F1 score = Harmonic mean of precision and recall score with range between 1 (best) and 0 (worst); AUROC = Area under receiver operating characteristic curve.

Model type	Accuracy score	Balanced accuracy score	Recall score	Precision score	F1 score	AUROC score
Logistic regression	**0.94**	**0.93**	**1.00**	**0.91**	**0.95**	**0.93**
Decision tree	0.53	0.51	0.60	0.60	0.60	0.51
Support vector machine	0.88	0.88	0.90	0.90	0.90	0.88

**Figure 3.**
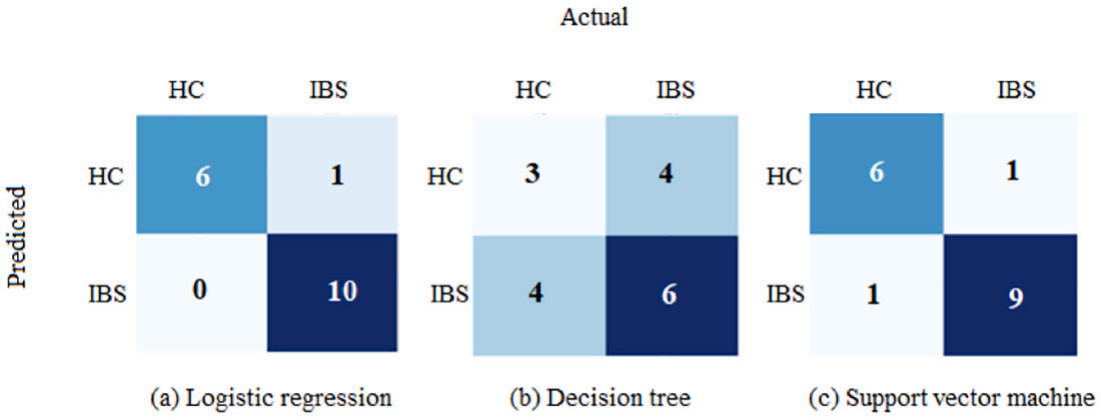
Classification of IBS and HC in the unseen test dataset (n = 17) using three different machine learning models. IBS = Participants with irritable bowel syndrome; HC = Healthy control subjects.

The LR and SVC models resulted in balanced accuracy scores of 0.93 and 0.88 respectively. The overall best evaluation metrics were noted for LR model, such as a recall score of 1 and precision score of 0.91. Similarly, F1 score, which refers to weighted harmonic mean of precision and recall and AUROC score were also highest for LR model. On contrary to both LR and SVC models, the decision tree (DT) model performed poorly on test set as its balanced accuracy score (0.51) was not only substantially lower than the other models applied on test set but was also significantly lower than the balanced accuracy of the same DT model applied on validation data (0.88). The LR model correctly classified all participants with IBS and 6 out of 7 HC subjects. The SVC model correctly classified 9 out of 10 patients with IBS and 6 out of 7 HC subjects. A significant number of misclassifications can be noticed for underperforming DT model, which correctly classified 6 out of 10 patients with IBS and 3 out of 7 HC subjects. The performances of these classifiers are also shown using confusion matrices in Figure 3.

### Contribution of psychological and fatigue symptoms to identify the presence of IBS

**Figure 4.**
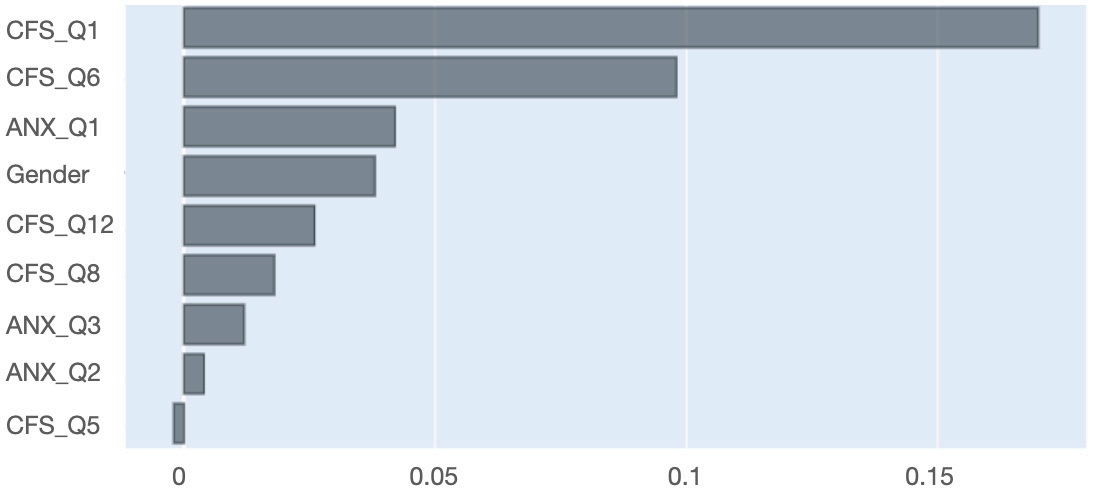
Feature contribution for the logistic regression model using permutation feature importance. CFS_Q1. = First question in Chalder Fatigue Scale; CFS_Q6 = Sixth question in Chalder Fatigue Scale; ANX_Q1 = First question in anxiety questionnaire; Gender = Participant gender; CFS_Q12 = Twelfth question in Chalder Fatigue Scale; CFS_Q8 = Eighth question in Chalder Fatigue Scale; ANX_Q3 = Third question in anxiety questionnaire; ANX_Q2 = Second question in anxiety questionnaire; CFS_Q5 = Fifth question in Chalder Fatigue Scale.

The LR model, which showed highest balanced accuracy score and outperformed other comparable models, used a feature set comprising of a combination of nine input features. Three of these symptom features were taken from the anxiety subscale (i.e. ANX_Q1, ANX_Q2, ANX_Q3), five features from the fatigue questionnaire (i.e. CFS_Q1, CFS_Q5, CFS_Q6, CFS_8, CFS_Q12), and gender as an add-on feature. The contributions of these features in driving the decision of the model using permutation feature importance method are shown in Figure 4. A detailed description of these features is given in Table 5.

**Table 5.** A list of symptom features used by the best performing logistic regression model to classify between irritable bowel syndrome (IBS) and non-IBS cases. ANX = Anxiety questionnaire; CFS = Chalder fatigue scale.

Feature	Description	Item response
ANX_Q1	I feel tense or wound up	i. Most of the time
		ii. A lot of time
		iii. Time to time
		iv. Not at all
ANX_Q2	I get a sort of frightened feeling like	i. Very definitely and quite badly
	something awful is about to happen	ii. Yes, but not too badly
		iii. A little, but it does not worry me
		iv. Not at all
ANX_Q3	Worrying thoughts go through my mind	i. A great of time
		ii. A lot of time
		iii. From time to time
		iv. Only occasionally
CFS_Q1	Do you have problems with tiredness?	i. Much more than usual
		ii. More than usual
		iii. No more than usual
		iv. Less than usual
		v. Not at all
CFS_Q5	If you have felt tired, for how long	i. Sixth months or more
	have you felt tired?	i. Between three and sixth months
		iii. Less than a month
		iv. Less than a week
		v. I don’t feel tired
CFS_Q6	Do you lack energy?	i. Much more than usual
		ii. More than usual
		iii. No more than usual
		iv. Less than usual
		v. Not at all
CFS_Q8	Do you have less strength in you muscles?	i. Much more than usual
		ii. More than usual
		iii. No more than usual
		iv. Less than usual
		v. Not at all
CFS_Q12	Do you have difficulties in concentrating?	i. Much more than usual
		ii. More than usual
		iii. No more than usual
		iv. Less than usual
		v. Not at all
Gender	What is your gender?	i. Female
		ii. Male

A post-hoc LOOCV analysis using LR also established the significance of nine item feature set, when applied on complete 84 participant dataset i.e. balanced accuracy score 0.84, precision 0.86 and recall 0.88. These performance metrics are slightly inferior but comparable to metrics achieved from evaluation of model on test set.

## Discussion

In this study, we have developed three machine learning models to identify psychological, fatigue and sleep related symptoms from four questionnaires that are of particular importance in describing the syndrome of psychological symptoms in patients with IBS.

The balanced accuracy scores for all machine learning models on the validation dataset ( Table 3) are generally above 0.75 irrespective of the questionnaire group used for feature selection or the type of the model applied. In a few instances, however, these scores were lower than 0.75 and a minimum accuracy of 0.69 was noted in one instance only. This shows high relevance and significance of selected questionnaires in IBS, which aligns well with results from previous studies ^1– 10^. The integration of various questionnaires in feature selection generally improved model accuracy (mean accuracy 0.81) compared to the instances where the individual questionnaires (mean accuracy 0.78) were used in feature selection. This pattern likely indicates complementarity of information provided by grouping various questionnaires together as it led to superior discriminability between IBS and HC groups.

However, the integration of more questionnaires did not always improve model performance. The best performing LR model ( Table 3), with a validation score of 0.86, used a combination of items only from the anxiety subscale from HADS and the fatigue questionnaire and outperformed the comparable LR model that used features from all questionnaires (accuracy of 0.83). On the contrary, the SVC model showed higher accuracy (0.93) when a group of all questionnaires was used. These results likely indicate the presence of a complex underlying multivariate association between IBS pathophysiology and co-existing psychological symptoms, as previously pointed out in conclusions from previous studies ^15, 16^. In addition, it shows that IBS and HC groups can be separated from each other by using a variety of combinations of questionnaires and their associated symptom features. Such a selection is, however, sensitive to the type of the model used.

Out of all three best performing models, both the LR and SVC models maintained their superior performance metrics when tested on an unseen test dataset and resulted in balanced accuracy scores of 0.93 (95% CI, 0.81 − 1.0) and 0.88 (95% CI, 0.72 − 1.0), respectively. This further endorses the importance of symptoms of anxiety, depression, fatigue and sleep when classifying a patient with IBS, and indicates that a machine learning based evaluation can contribute to improve this classification in a clinical setting. However, this essentially requires further validation of the model on larger datasets acquired from patient groups with broader demographic characteristics. The DT model, however, performed poorly on the test set and resulted in balanced accuracy of 0.51 (95% CI, 0.29 0.76). This drop in performance may be explained by potential overfitting of the model, compounded with limited sample size in training phase, as decision trees are intrinsically prone to over fit the training data. On the basis of its performance on unseen test set, the LR model is nominated as the most suitable model for evaluation of patients with IBS in our cohort.

Out of multiple IBS co-morbidities assessed in this study, anxiety and fatigue stood out as the most significant problems associated with IBS as the final LR model only used symptom features related to these disorders. This clearly suggests that evaluation of anxiety and fatigue related symptoms should be an integral part in IBS assessment, which also answers the first question posed in the introduction section. The presented study also provides some indications about the items that should be selected i.e., the second question. This was shown by the feature set included in the best performing model and their feature ranking scores. As illustrated in Figure 4 and Table 5, the final feature vector comprises a total of nine features, where five are related to fatigue (CFS: Q1, Q5, Q6, Q8, Q12), three are related to anxiety (ANX: Q1, Q2, Q3) and one is the patient’s gender. Among anxiety and fatigue, fatigue was associated with superior discriminability on the basis of (i) higher number of fatigue related symptoms in the feature vector and (ii) their higher contribution in terms of permutation testing scores: the first two most contributing symptoms come from fatigue questionnaire, with a cumulative contribution score of about 25%. On the other hand, the cumulative contribution score of all anxiety related symptoms is less than 10%. The machine learning models also eradicated the need to manually define optimal cut off values (Question 3) and aggregation of selected features into total scores (Question 4), as relative weight of each selected feature was learned in the model training phase and formed the basis for the decision to classify participants in either IBS or HC category.

A combination of fatigue and anxiety related features in driving an accurate classification output of the model, indicates the presence of multivariate associations of more than one syndrome of psychological symptoms in patients with IBS. The contribution of gender in our model as an input parameter aligns well with the existing literature, as its significance in IBS is well-established ^30^. It is also important to note that permutation feature testing does not necessarily represent intrinsic predictive value of a feature but only describe its importance with respect to a particular classifier.

The presented study has several limitations. Firstly, the machine learning models were developed, validated and tested on a rather small dataset of 84 participants only. This can be attributed to limited enrolment of IBS and HC participants in our patient cohort within the allowed time and resources. Although, our modelling approach resulted in high accuracy models from this data, it is important to note that all of our performance estimates are based on small validation and test sets (n = 17). It is also important to note that despite the limited sample size of our study, the development of reliable machine learning models is achievable for a sample size of more than 50 participants, as advised in the scikit-learn machine learning library guidelines ^31^. In addition, the outcomes of our models were supported by Leave-One-Out Cross-Validation analysis ^32^. Limited sample size also restricted development of gender specific models for male and female participant groups, which could further elaborate the role of gender in evaluating psychological status in IBS.

Prior to practical application of the proposed model, its validation and testing on larger unseen datasets is necessary. Similarly, using additional data to re-train the same model using the approaches proposed in this study will also be useful. Secondly, the dataset was significantly imbalanced as the number of females and participants with IBS was substantially higher than the male participants and HC group, respectively. This factor is also attributed to the availability of participants to enroll in our study. However, the underrepresentation of any one class in the dataset may affect the rules of learning in the training dataset. For example, if a certain gender group is dominant in the participant cohort in terms of number of participants and it also belong to the IBS category, the models may learn an unwanted association of that gender with IBS i.e. a decision rule that may not necessarily represent the reality but instead originates from gender class imbalance. Any future studies and extension of the presented work will essentially benefit from datasets with balanced representation of classes. Thirdly, the inclusion criteria of the IBS group in our study required a score greater than 175 on IBS symptom severity scale (IBS-SSS) i.e. moderate or severe IBS ^28^. Hence, the effectiveness of the proposed model in classifying patients with mild IBS i.e. IBS-SSS score less than 175, might be limited and future studies should consider covering the complete range of IBS-SSS score scale. Fourthly, the dataset was collected from a certain region in Norway and only represented a small demographic segment of broader IBS population. The association between psychological symptoms and IBS pathophysiology may vary with respect to demographic attributes and genetic background, which can potentially limit the application of the proposed models to a given region only. This issue can be addressed through data collection from a multi-center research design performed in broader and diverse demographic regions.

## Conclusions

To conclude, we proposed a new psychological symptoms based evaluation system using machine learning methods to complement clinical diagnosis of IBS. Our final psychological model for IBS has shown high accuracy when tested on an unseen test set. In addition, various items from fatigue and anxiety scales were found to be the most significant as they drove highly accurate classification decisions. This combination of items likely indicates the presence of a complex multivariate association between IBS pathophysiology and symptoms associated with fatigue and anxiety. However, due to the limited sample size in our study, the developed model requires broader validation and testing prior to clinical application. In future studies, the predictive performance and generalization of the proposed symptom based models may be further improved with integration of broader biological data e.g. microbial, metabolic, hormonal, immunological and likely also neuroimaging-derived biomarkers.

## Ethics and consent

The Bergen Brain Gut study was performed in accordance with the ethical requirements defined in the Declaration of Helsinki and approved by Regional Ethical Committee (approval no. 2015/1621/REK). All participants gave their informed consent prior to their inclusion in the study.

## Data Availability

Zenodo: ORE_Psych_ML_IBS https://zenodo.org/record/7454380 (Lundervold, 2022) This project contains the following underlying data: 10fold_results_DT.pickle13.0 kB 10fold_results_LR.pickle13.0 kB 10fold_results_SVC.pickle13.0 kB pq_df_pt_anon31.0 kB pq_df_pt_anon.csv15.5 kB testing.csv3.2 kB train_valid_test.csv14.2 kB train_valid_test_TFS_Chalder.csv14.6 kB training.csv8.6 kB validation.csv3.2 kB Data are available under the terms of the Creative Commons Attribution 4.0 International license (CC-BY 4.0).

## Software availability

Source code available from: https://github.com/MMIV-ML/ORE_Psych_ML_IBS
