# Supplementary material for: A psychological symptom based machine learning model for clinical evaluation of irritable bowel syndrome
Source: Open Res Eur. 2023 Jan 27;3:19. [Version 1] doi: 10.12688/openreseurope.15009.1 (PMC10457559; doi:10.12688/openreseurope.15009.1)
Supplement: Peer review history

# Peer review: Reviewer response for version 1

**DOI:** 10.21956/openreseurope.16227.r34096

**Published:** 2023-08-25

**Authors:** Alexandre Denadai-Souza

**Affiliations:** 1 Associatie KU Leuven, Leuven, Flanders, Belgium

The authors developed a machine learning workflow based on 3 different models (i.e. logistic regression, decision trees, and support vector machine classifiers) to select the most significant psychological features associated with IBS. As highlighted by the authors, the logistic regression model performed the best. Of note, the most exciting finding derived from this model was the combinatorial association of anxiety and fatigue, but not depression, with IBS. This is intriguing considering that similarly increased odds for either anxiety or depression have been reported in patients with IBS (Zamani *et al.*, 2019 ^1^). Therefore, future investigations with bigger sample sizes will be instrumental to confirm or refute these findings. Besides the higher prevalence of anxiety and depression in patients with IBS, several studies have also indicated higher levels of these psychological disorders in patients with IBS in relation to subjects not suffering from this functional gastrointestinal disorder. It remains unclear though how well the model will predict IBS once applied to a more heterogeneous population. Overall, the manuscript is well-written, and the outcomes are sound. In the methods section, more information should be provided regarding the inclusion/exclusion criteria for healthy volunteers and for the diagnosis of IBS.

 Minor comment:

 In Table 1, the percentage of males and females in each group should be reviewed.

Is the study design appropriate and does the work have academic merit?

Yes

Is the work clearly and accurately presented and does it cite the current literature?

Yes

If applicable, is the statistical analysis and its interpretation appropriate?

Yes

Are all the source data underlying the results available to ensure full reproducibility?

Yes

Are the conclusions drawn adequately supported by the results?

Yes

Are sufficient details of methods and analysis provided to allow replication by others?

Partly

Reviewer Expertise:

Pathophysiology of gastrointestinal disorders

I confirm that I have read this submission and believe that I have an appropriate level of expertise to confirm that it is of an acceptable scientific standard.

## References

[1] : Systematic review with meta-analysis: the prevalence of anxiety and depression in patients with irritable bowel syndrome. *Aliment Pharmacol Ther* .2019;50(2) : 10.1111/apt.15325 132-143 10.1111/apt.15325 31157418

# Peer review: Reviewer response for version 1

**DOI:** 10.21956/openreseurope.16227.r30745

**Published:** 2023-02-10

**Authors:** Shah Nawaz

**Affiliations:** 1 Deutsches Elektronen-Synchrotron DESY, Hamburg, Germany

**Summary:**

 The paper presents three machine learning models to identify psychological, fatigue and sleep related symptoms from four questionnaires in describing the syndrome of psychological symptoms in patients with Irritable bowel syndrome.

**Strengths:**

- Data collection and management is explained in details along with the method to handle missing information. This aspect was helpful for the reviewer to understand the data. Moreover, the data imbalance problem was also discussed in detail.
- The study also considered ethics while developing methods for such clinical study
- Overall the paper is well written with appropriate information to understand the background for a machine learning expert.

**Weaknesses:**

- Ethics related to machine learning must also be discussed. Some discussion related to explainability must be added.
- The motivation is not clear why these specific three models were selected for the study.

Is the study design appropriate and does the work have academic merit?

Yes

Is the work clearly and accurately presented and does it cite the current literature?

Yes

If applicable, is the statistical analysis and its interpretation appropriate?

Yes

Are all the source data underlying the results available to ensure full reproducibility?

Yes

Are the conclusions drawn adequately supported by the results?

Yes

Are sufficient details of methods and analysis provided to allow replication by others?

Yes

Reviewer Expertise:

Computer vision and machine learning. The reviewer is working on combing multiple modalities such as image/text or audio/image for various computer vision tasks.

I confirm that I have read this submission and believe that I have an appropriate level of expertise to confirm that it is of an acceptable scientific standard.
